# EANM procedural recommendations for managing the paediatric patient in diagnostic nuclear medicine

**DOI:** 10.1007/s00259-023-06357-3

**Published:** 2023-08-09

**Authors:** Luca Camoni, Andrea Santos, Marie Luporsi, Ana Grilo, Agata Pietrzak, Jonathan Gear, Pietro Zucchetta, Zvi Bar-Sever

**Affiliations:** 1 University of Brescia, 25123 Brescia, Italy; 2 Nuclear Medicine Department, University of Brescia, ASST Spedali Civili Di Brescia, P.Le Spedali Civili 1, 25123 Brescia, Italy; 3 Nuclear Medicine Department, CUF Descobertas Hospital, Lisbon, Portugal; 4 Department of Nuclear Medicine, Institut Curie, PSL Research University, 75005 Paris, France; 5 LITO Laboratory INSERM U1288, Institut Curie, 91440 Orsay, France; 6 H&TRC — Health and Technology Research Center, ESTeSL — Escola Superior de Tecnologia da Saúde, Instituto Politécnico de Lisboa, Lisbon, Portugal; 7 CICPSI, Faculdade de Psicologia, Universidade de Lisboa, Alameda da Universidade, Lisbon, Portugal; 8 Electroradiology Department, Poznan University of Medical Sciences, Poznan, Poland; 9 Nuclear Medicine Department, Greater Poland Cancer Centre, Poznan, Poland; 10 Joint Department of Physics, Royal Marsden Hospital and Institute of Cancer Research, Sutton, UK; 11 Nuclear Medicine Department, Padova University Hospital, 35128 Padua, Italy; 12 Department of Nuclear Medicine, Schneider Children’s Medical Center, Tel-Aviv University, Petach Tikva, Israel

**Keywords:** Paediatric, Nuclear medicine, Patient management, Exam optimization, Psychology

## Abstract

**Purpose:**

The manuscript aims to characterize the principles of best practice in performing nuclear medicine procedures in paediatric patients. The paper describes all necessary technical skills that should be developed by the healthcare professionals to ensure the best possible care in paediatric patients, as it is particularly challenging due to psychological and physical conditions of children.

**Methods:**

We performed a comprehensive literature review to establish the most relevant elements of nuclear medicine studies in paediatric patients. We focused the attention to the technical aspects of the study, such as patient preparation, imaging protocols, and immobilization techniques, that adhere to best practice principles. Furthermore, we considered the psychological elements of working with children, including comforting and distraction strategies.

**Results:**

The extensive literature review combined with practical conclusions and recommendations presented and explained by the authors summarizes the most important principles of the care for paediatric patient in the nuclear medicine field.

**Conclusion:**

Nuclear medicine applied to the paediatric patient is a very special and challenging area, requiring proper education and experience in order to be performed at the highest level and with the maximum safety for the child.

## Preamble

The European Association of Nuclear Medicine (EANM) is a professional non-profit medical association that facilitates communication worldwide among individuals pursuing clinical and research excellence in nuclear medicine. The EANM was founded in 1985.

These recommendations are intended to assist practitioners in providing appropriate nuclear medicine care for patients. They are not inflexible rules or requirements of practice and are not intended, nor should they be used, to establish a legal standard of care.

The ultimate judgment regarding the propriety of any specific procedure or course of action must be made by medical professionals taking into account the unique circumstances of each case. Thus, there is no implication that an approach differing from the recommendations, standing alone, is below the standard of care. To the contrary, a conscientious practitioner may responsibly adopt a course of action different from that set out in the recommendations when, in the reasonable judgment of the practitioner, such course of action is indicated by the condition of the patient, limitations of available resources, or advances in knowledge or technology subsequent to publication of the recommendations.

The practice of medicine involves not only the science but also the skills to deal with the prevention, diagnosis, alleviation, and treatment of disease.

The variety and complexity of human conditions make it impossible to always reach the most appropriate diagnosis or to predict with certainty a particular response to treatment. Therefore, it should be recognized that adherence to these recommendations will not ensure an accurate diagnosis or a successful outcome.

All that should be expected is that the practitioner will follow a reasonable course of action based on current knowledge, available resources, and the needs of the patient to deliver effective and safe medical care. The sole purpose of these recommendations is to assist practitioners in achieving this objective.

## Introduction

Diagnostic nuclear medicine (NM) includes a wide variety of procedures that can be applied to adult and paediatric patients. Management in paediatric patients requires a special set of skills, which should be developed by the nuclear medicine professionals who are involved in the procedures. NM teams face several challenges while performing diagnostic procedures on the youngest population, as many technical details and requirements may change according to the patient’s physical characteristics (e.g., weight or body mass index) as well as behavioral and psychological aspects related to the stage of development [1]. An advanced understanding of how to adapt to each paediatric patient includes the ability to optimize the administered activity and acquisition details for each patient to deliver the lowest possible dose, while attaining a good quality image. Additionally, the capability of communicating effectively with the child and the caregivers is crucial. Strategies for relieving anxiety and promoting patient cooperation are essential for best practice [2]. Developing the communication ability and the capacity to better understand the needs of each patient in each patient’s stage of development will enable the application of a personalized approach [3]. Having a more relaxed patient and caregiver helps in reducing many common image artifacts, such as patient motion, while increasing comfort for both the patient and caregivers. Tailoring the exam to the paediatric patient requires consideration at each step of the process, from scheduling the scans to the final words, before children leave the department.

### Goals

The purpose of this EANM procedural recommendation is to highlight best practices by identifying strategic options for patient compliance and dose optimization in the NM technologist’s interaction with children throughout all stages of the imaging procedure.

## Scheduling and preparations

Every nuclear medicine examination has to be justified and optimized according to the ALARA principle (“As Low As Reasonably Achievable”) [4]. In paediatric nuclear imaging, one of the best allies for optimization remains anticipation.

### Scheduling

After having validated the indication, relevance, and feasibility of NM imaging (principle of justification [5]; e.g., *Is this the most suitable test? Is the patient physically able to undergo the scan? Are the technical and human resources sufficient?*), attention should be paid to scheduling that is compatible with the required procedure preparation (e.g., scheduling examinations that require fasting early in the morning). Avoiding delays in paediatric exams and excessive waiting times should guide the scheduling procedure [6]. Consideration of the department’s workload (including the availability of the team in its full capacity) should also be taken into account as caring for a child requires a lot of time, energy, and human resources. Limiting the time spent in the waiting room before the examination helps reduce the anxiety of children and increase their comfort [7].

It is useful to establish well-defined days for paediatric examinations and procedures carried out together with the paediatric and anesthetic department of the hospital and to ensure that qualified people are available on the day of the examination (paediatric nurse to easily find an intravenous access, especially in the very young children, senior anaesthesiologist available for performing general anesthesia when needed). It is important to ensure that all the medical equipment needed is available on the day of the nuclear medicine exam.

### Preparing for the examination day

Many aspects can be anticipated in order to prepare for the day of the examination and improve the patient and family experience in the NM department [2, 8]. Some parameters, such as the age of the child (neonate, 3 month–2 years, 2–5 years, 5–10 years, > 10 years), height, weight, medications (that might affect tracer distribution), and all the diseases and pathological conditions (autism, coma state, others), can be collected in advance and verified upon patient arrival in the department. Defining the acquisition protocol in advance enables proper scheduling of the department’s equipment with sufficient time for any necessary adjustments. Knowing what imaging is required and approximate duration helps to plan the time needed for each patient, avoiding delays that can cause additional stress and anxiety to the patient and the staff (e.g., three phase bone scintigraphy vs late imaging bone scintigraphy).

Premedication and specific preparation (*anxiolysis*, *analgesia*, *sedation*, *thyroid blockage*, *special diet*, etc*.*) should also be anticipated and planned. If the department does not have personnel skilled in paediatric venipuncture, it is recommended that a dedicated team obtain intravenous access in advance whenever possible, especially for younger children. The team must recommend to the stakeholder responsible for the child to monitor his/her movements to prevent any motion that might disrupt the IV and keep it open until after the examination is complete.

**Fig. 1:**
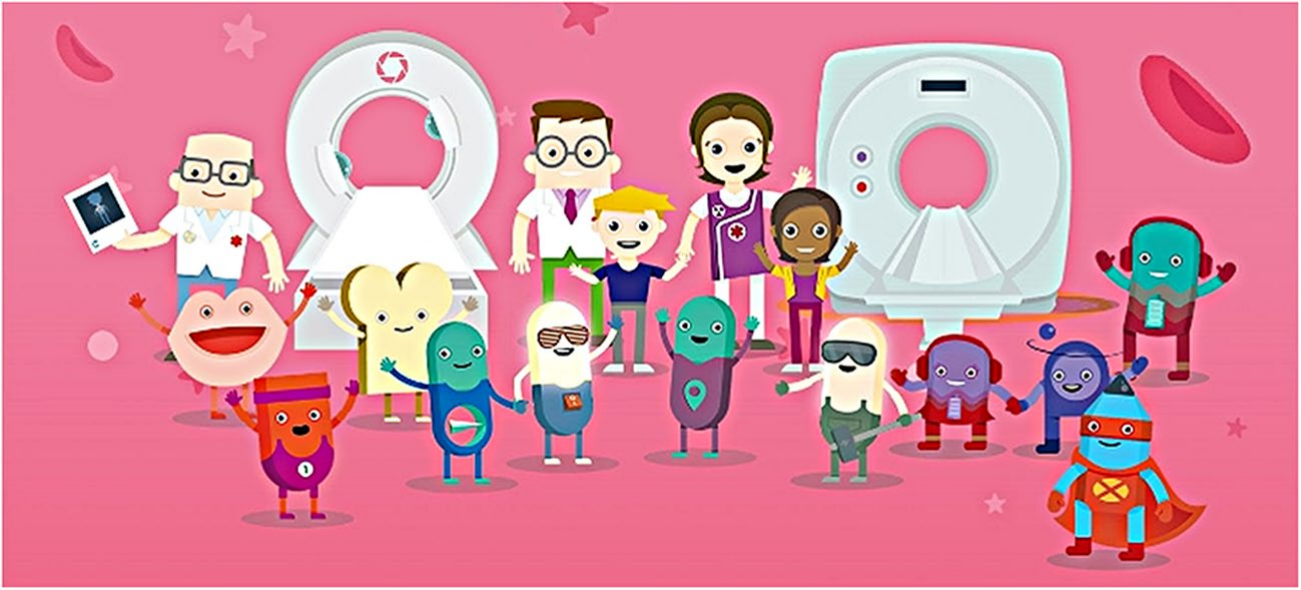
An image from Sunny the isotope and Tim. Dr Ronald Van Rheenen created the cartoon figures Sunny the isotope and Tim the pharmaceutical to explain nuclear medicine imaging to young children. Their adventures in the body and their encounter with Rob the receptor are presented in YouTube clips, books, and wall posters. Children are encouraged to learn about the characters and their adventures prior to their visit to the nuclear medicine department. Explaining the procedure to children according to their level of comprehension is effective in reducing anxiety and improving cooperation. (https://www.eanm.org/sunny-tim/)

Clear and concise instructions should be provided concerning the procedure and its preparation to the family and care team, both orally and in writing (also using illustrated booklets) [9, 10]. In a study related to paediatric [^99m^Tc]Tc-DMSA-scintigraphy, Train et al. [11] showed that psychological preparation, including a photo booklet and a letter providing advice to parents, sent a week before the appointment decreased children’s distress and the need for sedation. Illustrated booklets [11, 12] or animated educational videos that parents can watch with their child (Fig. 1) [13, 14] can be beneficial for the communication with both adults and children [15]. These tools allow the child to become familiar with the scanner and better understand what will happen and what they will have to do in a playful way [11]. These tools should be appropriate to the age of the child [9] and be provided to the child with the necessary lead time. However, with younger children, the disclosure should be close to the exam date because if it is several days before, it can increase anxiety.

Supplying written information and providing further recommendations directed to caregivers is also essential [9, 11]. The complexity of NM examinations can confuse caregivers; therefore, enough time must be given to understand the procedure and to answer caregivers’ questions regarding all possible aspects they had not previously considered [15]. In this sense, during scheduling, it is crucial to offer contact details (email, phone) for caregivers to raise any doubts or concerns they might have. Additionally, practical information, such as the importance of bringing the child’s emotional and transitional object, if appropriate, and having objects or games that will help the child be distracted and remain still, should be included in the written information [11]. It is essential to ensure that pregnant mothers are aware of any restrictions regarding their attendance at the department; for instance, they cannot accompany their child as a sole caregiver, and this must be communicated clearly.

## Welcoming, explaining, and cooperating

### Welcoming

**Fig. 2:**
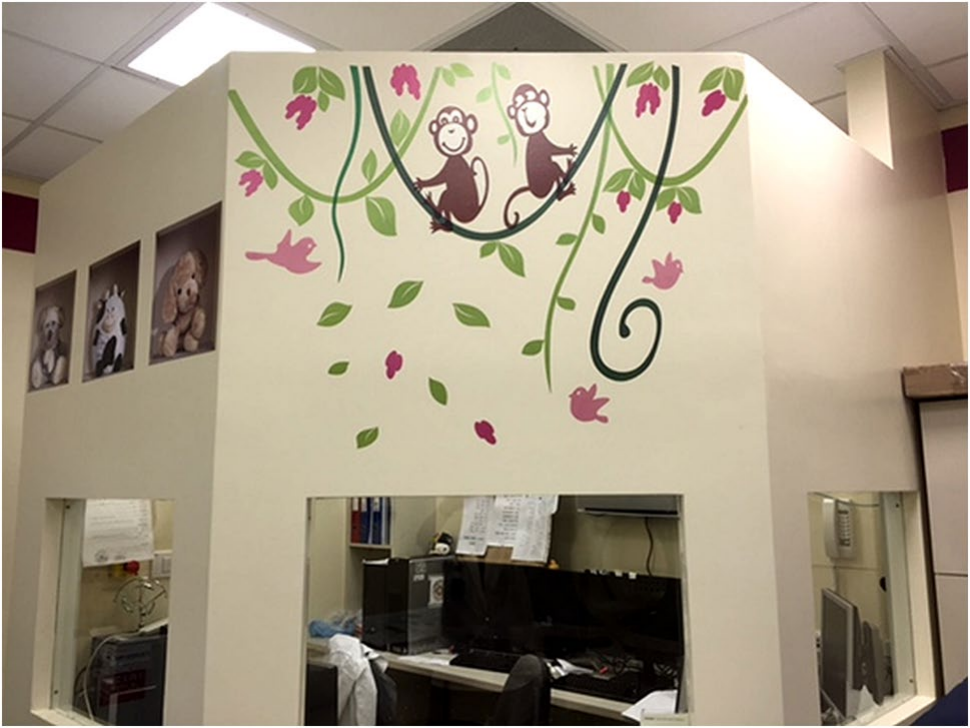
Example of wall decorations, they are important for creating a child-friendly atmosphere in the nuclear medicine department. (From Schneider Children’s Medical Center, Israel)

Upon arrival, patients should find a welcoming and child-friendly atmosphere [8] where nuclear medicine professionals show genuine interest in the needs and concerns of the child and caregivers. Specific environmental strategies directed at a comfortable ambience can help in limiting the anxiety (e.g., in the waiting area). Waiting rooms should be designed using welcoming decorations and drawings (Fig. 2). It is essential to entertain and distract children in the waiting room, especially due to prolonged waiting times between tracer administration and imaging, required for certain studies (e.g., bone scintigraphy and DMSA scans). Audio-visual tools can be considered (videos, music), and/or allowing children to bring their own toys from home. Using the child’s own toys is preferable as it ensures that the available entertainment objects are personalized according to their preferences. Additionally, board games or toys can be offered in the paediatric waiting room to help ease the waiting period. These items should be divided into different age groups, allowing for selection prior to arriving in the paediatric waiting room. It is important to avoid games with small parts that pose a choking hazard risk, and great attention should be paid to ensure that the toys are washable and can be decontaminated after each use. Such solutions may help to indicate the empathy and involvement of the healthcare team. The duration of preparing for the study should be kept to a minimum, and separate dedicated paediatric waiting rooms should be available if feasible [2, 7, 10, 12, 16]. To establish a relationship of trust and to minimize child’s anxiety about the unknown, the child and caregivers should only be approached by the NM professionals essential for the procedure [9].

It is important not to assume that the child and his caregivers are fully aware of the study procedure. Upon entering the nuclear medicine department, the child and the accompanying person (*parents or caregiver*) may or may not have a clear understanding of the imaging process, and this idea may or may not be correct. Referring physicians can sometimes lack the knowledge required to precisely describe the imaging procedure to the patient and caregivers [17, 18]. The child is often worried about any pain that he/she might experience [19]. Caregivers are often more worried than the child [9]. They are more likely to be concerned about the exposure to radiation and possible adverse effects throughout child’s lifespan [20], the complexity of the imaging procedure, and the time to obtain scan results. All caregivers should all be encouraged to ask any question or discuss any concerns they have. Communication is a major tool for the successful completion of the procedure [2, 9, 21]. Moreover, verbal and non-verbal communication are both proven to be effective in reducing anxiety [2, 9, 22, 23]. Parents anxiety is a powerful predictor of procedural anxiety in children [9, 24, 25]. Therefore, NM professionals should be sensitive and avoid criticizing caregivers’ anxiety and difficulties in calming the child; instead, they must use a family-centered communication approach, promoting caregivers’ engagement and proposing straightforward strategies in a friendly way.

It is essential that caregivers feel valued and engaged during all the NM procedures with the child [9]. Active listening, openness (e.g*.*, *Do you have any doubtd /is something upsetting you? I noticed that you have been to the NM department before. How was it*? *Is there anything specific you want me to know about?*) and closed questions (e.g., *Did you have get the chance to read the* *leaflet we sent to you by email*? *Is this your child’s first time in the NM department?*) are effective communication techniques [25].

### Explaining the procedure and encouraging cooperation

A clear and concise description of the exam, offering detailed procedural and sensory information in an age-appropriate manner, helps to minimize the stress, by reducing uncertainty and unknown and improving the child’s and caregivers’ experience and cooperation. The caregivers must obtain clear instructions on what is required of the child during the examination and what they may do to support them [2]. Providing understandable information empowers children and caregivers to cooperate during the procedure [25]. The child knows exactly what will happen and how he/she should behave, and caregivers realize their role and how to support the child. Consequently, the healthcare professionals need to be able to provide all necessary instructions, to answer urgent questions, and to address all possible concerns that can occur during the procedure. Clear and plain language improves communication [25]. Improved communication skills can be acquired through training [2, 26], and with a bit of experience, the technologist will become more comfortable in appropriately communicating and reassuring both the patient and his/her entourage [2, 26].

Communication should be guided by the developmental level of the child [8]. Based on Piaget’s cognitive development theory, Bibace and Walsh [27] describe six developmental categories of explanations of illness that are extremely valuable for communication with the child (*phenomenon* and *contagion* related to pre-school age, *contamination* and *internalization* related to school age, and *physiological* and *psychological* associated with adolescents). Knowledge of these stages is fundamental to understand children’s perceptions of health-related events and information-processing capabilities, as children have specific medical procedures, needs, perceptions, and worries at each stage [28]. For example, pre-school children do not understand the reason for the NM procedure, because they have concrete thinking founded on a perceptive basis and little understanding of the functioning of the human body and are unaware of most of the internal organs. Therefore, information should focus on what the child must do and/or will feel (not the examination itself), using simple language and appealing to visible parts of the body (e.g., *Let’s take some special pictures of your belly. Do you want to play the statue game? You must be very still.*). Simulations before the examination and the notion that the caregiver will always be with them benefit pre-school children.

With school-age children, the provision of information can be more complete and more realistic, as these children have logical thinking about actual events and begin to know more about human organs. In a study with children between 8 and 12 years old, Bray, Appleton, and Sharpe [29] have shown that children of this age value preparation for procedures and identify three types of information: procedural information (*What will happen?*), sensory information (*Will I feel scared?*), and self-regulation information (*What can I do to stay calm?*). Therefore, NM procedures should be explained based on analogies from the child’s world (e.g., It is like a photograph. You cannot move; otherwise, it gets blurry or out of focus). The simple effects of exams highlight the physiological aspects (e.g., You’ll get a little sting on your arm. Then we will put on a great band-aid of your choice.). Concrete methods such as drawing, models, or videos effectively explain the procedure.

Cooperative behavior should be rewarded [8], and reinforcements must be presented when explaining the procedure to focus children’s attention on what they have to do to obtain them. Therefore, it must be indicated that if the child completes the whole procedure adequately, they will be entitled to a special gift.

The risk-to-benefit ratio of the procedure must be properly explained to the patient and caregiver by the healthcare team. The clinical consequences must be clearly outlined if the examination is refused: the missed diagnosis and the potentially delayed treatment can affect the patient’s management as a whole as well as the final medical outcome [2].

NM team must be particularly attentive to questions and concerns about ionizing radiation [30, 31]. Indeed, many parents worry about the radiation exposure and are afraid of possible side effects [32, 33]. Providing clear and concise information on dosimetry is one of the tasks of healthcare professionals as also emphasized by the World Health Organization (WHO) [34]. A common technique to explain the “amount” and risk of radiation that will be received during the procedure is to make comparisons with more recognizable sources of exposure (e.g., *period of exposure to natural background radiation*, *flight hours in air travel*, *number of chest X-rays*, *comparison with other risks faced in daily life such as car driving…*) [19].

The presence of the caregiver during the medical procedure should be encouraged as it reassures the child [9], potentially benefiting the final study outcome [35, 36], and often legally required [18, 37]. This collaboration not only may include the company of a caregiver but also provide positive distractors, such as favorite toys (brought by them or one that is provided by the NM unit and that must be washable and easy to decontaminate) and other objects important to the child. These objects can comfort the child during the procedure and may also be used to visualize and explain the procedure beforehand [2, 16]. To increase the collaboration also maintain a continuous conversation with the patient until the end of the procedure, furthermore any additional acquisitions or delays should be explained [18].

## Preparation, premedication, and injection

### Preparation and premedication

Discussions with the patient and the accompanying person must be concise but sufficiently thorough to collect certain essential information: weight and height, medical history and treatment sequences, list of medication, and available intravenous lines (peripheral or central catheter) [2, 8]. Particular attention should be focused on the observance of premedication and preparation (e.g., compliance with potassium iodide before injection of [^123^I]mIBG [38]). The possibility of pregnancy must be considered in all persons of reproductive capacity: it must be carefully researched during the discussion and ruled out, in case of doubt, by a urine pregnancy test before injecting radiopharmaceuticals. Each center should have clear written procedures in place directing the operator in how and when to ascertain the likelihood of pregnancy [4].

Depending on the examination, some premedication or preparations are required and must be verified on a case-by-case basis. The most common examples are listed below:

- *Hydration and micturition*: Most NM studies require hydration. Oral hydration is generally sufficient, but for certain debilitated patients and for fasting patients, IV hydration may be necessary [4]. Oral hydration may include breast or formula milk, plain water, or soft drinks. Fasting children for [^18^F]FDG-PET/CT scans can be offered plain water but not any liquids that may contain sugar [4]. Bladder voiding prior to single-photon emission tomography-computed tomography (SPECT) and PET acquisitions is important because it reduces artifacts related to radiotracer excretion, improves patient comfort, and lowers radiation exposure. In young children, urination can be stimulated by various methods: the sound of running water from a faucet or holding young children in an upright position or even encouraging them to walk can promote micturition. Emptying the bladder is essential in diuretic renal scans because a full bladder can slow drainage and increase the likelihood of vesicoureteral reflux episodes [39]. Draining the bladder by administration of diuretics or with bladder catheterization is rarely advised (*with the exception of some diuretic renal scans*) as bladder catheterization carries a risk of nosocomial infection and increases stress and discomfort [4]. Diapers should also be changed before image acquisition with pelvic-centered imaging performed first in a series of scans if necessary.
- *Specific preparations*: Many NM examinations require more specific preparations, which are described in detail in dedicated EANM Guidelines. A common example is thyroid blockade with a saturated solution of potassium iodide before any [^123^I]mIBG injection to prevent thyroid uptake of free iodide [38]. Another example concerns brown fat accumulation, which is very common in young patients and often hinders [^18^F]FDG-PET interpretation. Brown fat can be significantly reduced by maintaining a warm room temperature during the uptake phase or providing a warm blanket; premedication with oral propranolol or diazepam is also effective [2, 8].

### Radiotracer injection

Adequate preparation of the child for the radiopharmaceutical injection is important because it can reduce the trauma perception and improve cooperation in future NM procedures [6]. To reduce pain and anxiety and for radiation protection purposes, intravenous access should be pre-planned whenever possible. Anesthetic cream (containing lidocaine and prilocaine) can be applied 1 hour prior to venous access [2, 8, 40]. If an intravenous cannula is already available, it should be used for tracer injection, and the line flushed with sufficient saline solution.

The venipuncture and injection are challenging for most children, so their cooperative behavior should be reinforced. Informing children that by keeping their arm still until the end of the injection, they will receive a gift (e.g., a balloon, a colorful sticker or for older children a badge indicating bravery) will comfort the children [41] and increase the likelihood of compliance, not only during the injection but also for the remainder of the procedure. The injection site should be monitored, especially in young children who cannot convey discomfort. Insertion of a secure peripheral intravenous catheter for tracer injection is advantageous especially in young children and infants. It reduces the risk of tracer extravasation and prevents any additional puncture (e.g., no extra puncture needed for furosemide administration during diuretic renal scans [2, 8]). For some children, needle-related procedures are very frightening and painful; in that situation, holding a caregiver or other adult’s hands could help, as squeezing the hand quite forcefully can help the child feel relief [16]. Using distraction techniques to draw the child’s attention from the injection is also an effective tactic.

Radiopharmaceuticals are particularly safe from a pharmacological perspective and are reported to be non-toxic, non-allergenic, and without adverse osmotic effect [42]. For these reasons, it is very easy to use them, even in the youngest [43, 44]. However, some radiopharmaceuticals do require specific precautions, which are reported both in the summary of product characteristics and in relevant guidelines. For example, [^123^I]mIBG has to be injected slowly (over 1 to 2 min), to avoid hypertension, nausea, or pallor (children with secreting tumor and a risk of hypertensive peak should be monitored during and after injection). Anaphylactic reactions have been rarely reported [38].

Injected activities should meet the ALARA principle [45]. The goal of dose optimization is to identify the amount of activity required to guarantee a satisfactory diagnostic examination with appropriate information content and quality, while maintaining reasonable imaging times and minimizing unnecessary radiation exposure.

Administered activity should follow the EANM dosage card in its latest version. This document was first published by the EANM in 2008 [46, 47]. Later, a consensus was reached with Society of Nuclear Medicine and Molecular Imaging (SNMMI) to harmonize activity guidelines between both groups [45]. In 2016, the last update of the EANM paediatric Dosage Card was issued. The Dosage Card is periodically updated [48].

The EANM also offers an online calculator (www.eanm.org/publications/dosagecalculator) as well as a smartphone application “Peddose” dedicated to clinical use.

## Positioning, immobilization, and distraction

### Positioning and immobilization

Keeping a child still and calm throughout an entire acquisition process can be challenging. The inability to move for a long time can generate significant anxiety and discomfort; moreover, younger children may not understand this requirement [3, 49]. This is where the distraction methods (see next section) play an important role.

**Fig. 3:**
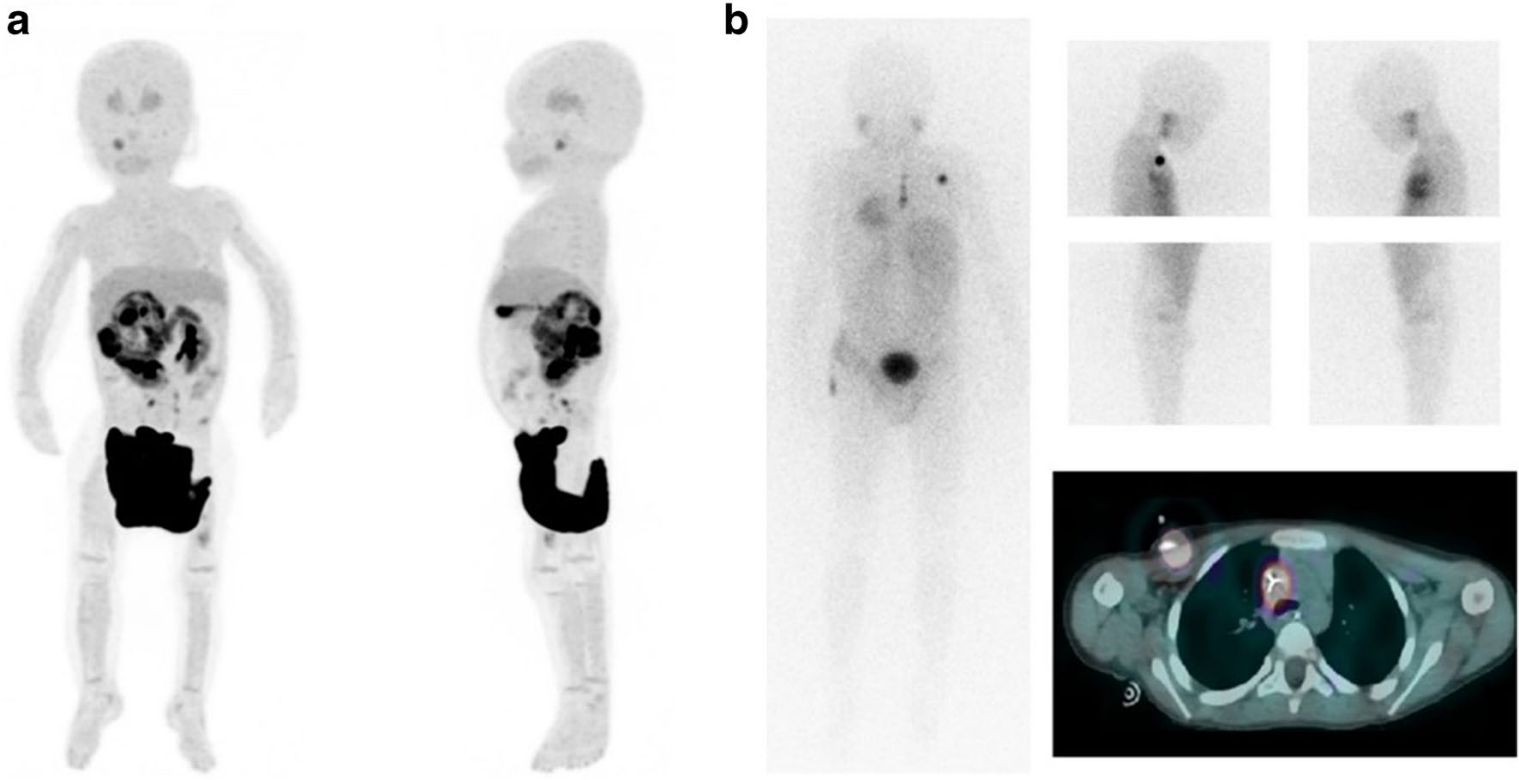
Two examples of imaging without diaper changing: **a** [^18^F]FDOPA-PET and **b** [.^123^I]mIBG with diaper and activity in the central catheter. If possible, changing the diaper before positioning the patient for PET or SPECT and planar imaging can improve image quality, avoid possible scatter-related artifacts, facilitate image interpretation, and decrease the need for further imaging and risk of contamination. (From Curie Institute, Paris, France)

**Fig. 4:**
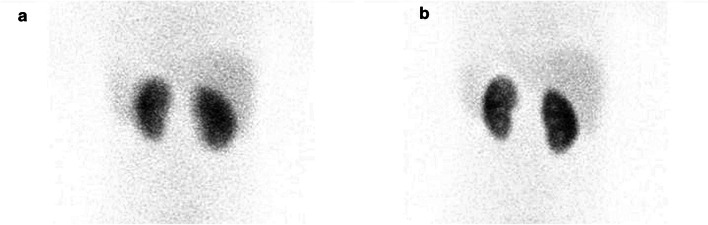
[^99m^Tc]Tc-DMSA renal cortical scintigraphy: static acquisition was performed with immobilization devices used, for a very energetic baby, slight movements of arms and legs (**a**). The same baby’s image was repeated after falling asleep (**b**). The image quality (sharpness) is considerably higher in the repeat acquisition

In most paediatric patients, imaging can be performed without any sedation. Optimal cooperation of the patient and parents is essential, and various approaches can be used depending on the child’s age [18, 36]. Diaper leaking must be avoided, and an adequately fastened, clean diaper is recommended before the exam (Fig. 3). Generally, the presence of a parent in the examination room is essential to reduce children’s anxiety and to improve cooperation. Some techniques can induce natural sleep in neonates and young infants. For example, parents should be instructed to prevent their infants from falling asleep while waiting for the scan (Fig. 4). Feeding them just before imaging, comfortably wrapping them up with a blanket on the camera bed and dimming the lights can often help to induce sleep. Proper positioning of the child is essential for a good quality image. Comfortable positioning and immobilization will reduce motion artifacts and consequently the total duration of the exam. Optimal positioning, with consideration given to symmetry of the studied areas, is essential for correct image interpretation. Furthermore, staff should know that it is normal for babies to cry during diagnostic examinations, and they should remain tolerant while ensuring that the baby is immobilized. Parents should not try to force the baby to stop crying, but should be informed that this is natural and acceptable, as the success of the examination depends mainly on the absence of movements; therefore, they can try to calm and comfort the baby and, if required, help the staff with the immobilization [50].

The term “immobilization” refers to a technique made with the consent of children and parents, as opposed to the term “restraint” where physical force is used to hold the child still during the imaging procedure [51].

**Fig. 5:**
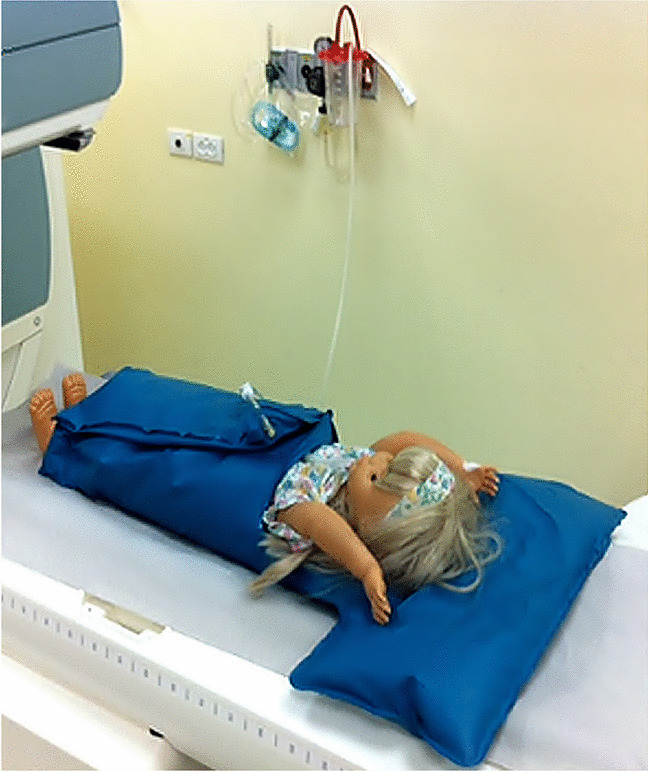
A vacuum mattress is an effective means to comfortably immobilize the child. This enables smooth acquisition without motion artifacts. (From Schneider Children’s Medical Center, Israel). The successful immobilization and positioning of the child requires several steps. Firstly, the diaper should be changed. Then, the infant should be placed on the examination table in a supine position with their arms along their body; this can be secured using bandage, sandbag, or headrest. The use of a comfortable position, restraint, and pacifier can help reassure the child and facilitate their induction into sleep

Comfortable immobilization devices exist [51, 52], such as vacuum mattresses (Fig. 5), cushions, sandbags, safety straps, or immobilization splints and pads. Whenever an immobilization device is used, it is mandatory to use it avoiding any airway obstruction or chest restriction and check respiratory excursions and the child’s head position frequently to guarantee airway patency. When the immobilization device is used, it is advisable to maintain an easy access to the patient’s body extremity in case of an emergency [53]. The devices used for patient comfort and immobilization should also be used mindfully so that no asymmetry is introduced through their use.

### Distraction techniques

**Fig. 6:**
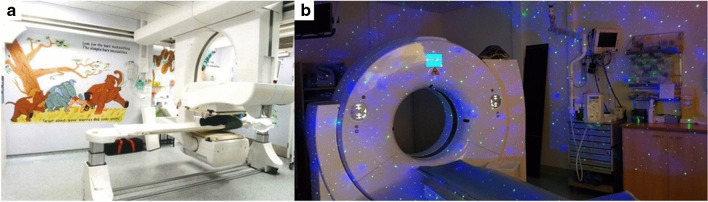
**a** “Jungle Book” images and song lyrics were painted on the walls of the gamma camera room by an artistic member of staff (from Royal Marsden Hospital, Sutton, UK). **b** Soothing ambient lighting reassures children and sometimes even helps them to fall asleep. Here, a transportable LED projector offers relaxing-colored atmospheres in a darkened examination room (From Curie Institute, Paris, France)

For older children, many effective distraction techniques can be employed. A child-friendly decor, an attractive, colorful, and stimulating environment can reduce fear and anxiety. Pictures, funny things, cartoons, or — for older children — challenges like riddles promote cooperation as children have things to look at that enable them to divert their minds away from the procedure [8, 16]. Some departments decorate their cameras with cartoon characters and stickers or use ambient lights in the examination room (Fig. 6).

**Fig. 7:**
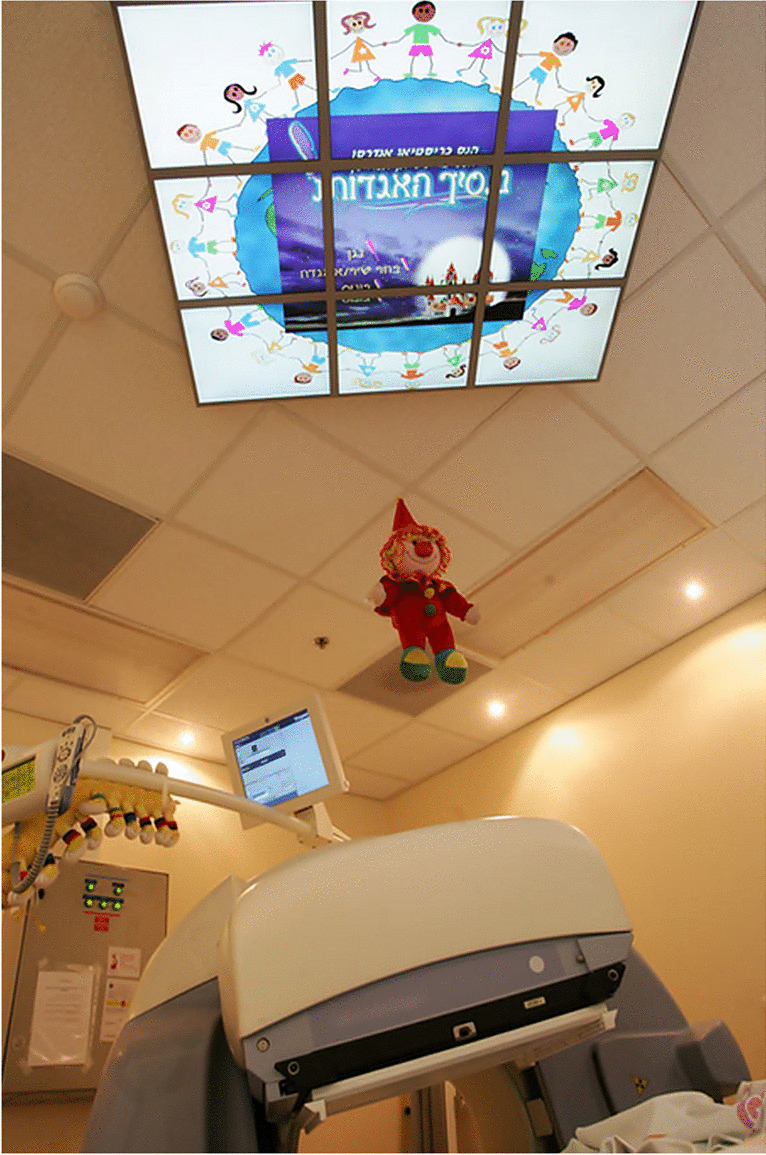
Audio-visual entertainment is highly effective in distracting children, reducing anxiety, improving cooperation, and minimizing motion during acquisition process. (From Schneider Children’s Medical Center, Israel)

Besides the environment, the child may be given the option to listen to music, storytelling, watch movies on a monitor (Fig. 7), or play games [2, 8]. Video goggles were found to be an effective distraction tool in school-aged children undergoing PET/CT [54]. A child’s focus on the distracting strategy could be increased if it is associated with a task (e.g., *In the end, you must tell me the colour of the sweater of the girl in the movie, OK? You must be watchful to see if a dog appears in the story that the mother is going to tell.*) [9]. The youngest children usually prefer parents to tell their favorite story, sing their favorite or familiar songs. These distraction techniques help children feel secure and assure them that they are not left alone in the examination room [2]. Some of these strategies need to be prepared when scheduling (e.g., bringing a favorite toy, story book, or video).

With pre-school-aged children, using distractive strategies should be mediated by caregivers (e.g., reading a story). At school age, children are less dependent on adults and more easily “engage on their own”, allowing them to concentrate longer on strategies such as watching a movie or reading a story. Nevertheless, caregivers should be involved to reinforce the distractive strategy the child is using [25]. Some older children and teenagers already have self-control strategies (e.g., *Try to think of something pleasant*) and do not consider acquiring images very demanding, managing to lie still without resorting to distracting strategies proposed by an adult [2].

Hypnosis and relaxation techniques (controlled breathing, imaginary, progressive relaxation, and even virtual reality) can also be considered. However, the first requires specific health-professional training.

## Anxiolysis, pain management, and sedation

### Anxiolysis

As previously discussed, communication, distraction, and relaxation techniques are very powerful anxiolytics, which are largely sufficient in most cases. However, the use of pharmaceuticals can sometimes be useful, and several options and administration routes exist [55]. Drug administration for pain management, anxiolysis sedation, and anesthesia should only be performed by authorized, qualified personnel according to hospital and national regulations (anesthetist or equally trained physician).

Inhalation of nitrous oxide is a practical option. It is an effective analgesic, anxiolytic gas with negligible side effects. However, nitrous oxide has limitations: A failure rate of 20 to 30% has been reported, and it is less effective in children under 3 years [56–58].

Hydroxyzine is a sedative antihistamine approved for anxiolytic use both in Europe and in the United States (US). It is available in tablets and syrup and has few contraindications in children [59].

Benzodiazepines, such as midazolam, requires a rectal or nasal administration and offers effective relief to anxious children. In some countries, it is widely used in paediatric imaging. However, benzodiazepines are most often not recommended in children under 16 years of age and in children in whom nitrous oxide is preferable [55, 60]. Combinations with other anxiolytics are contraindicated.

Other drugs of much less frequent use are also available such as intrarectal nalbuphine, sometimes used in combination with midazolam, or combination of intravenous drugs among fentanyl, midazolam, and ketamine [55, 60].

### Pain management

**Fig. 8:**
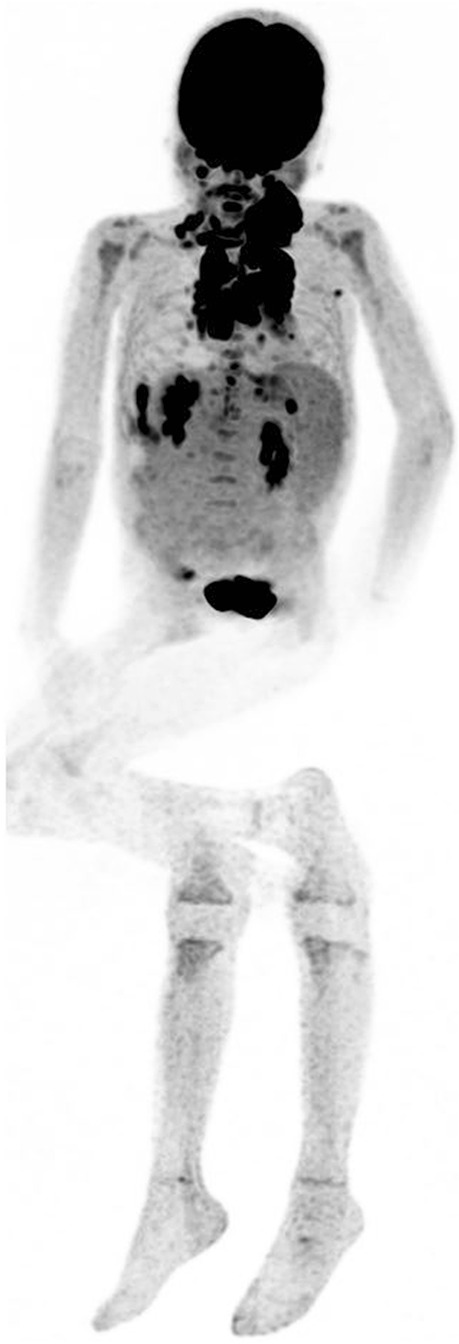
A child who is experiencing pain, is anxious, or uncomfortable is likely to move during acquisition. [^18^F]FDG-PET/CT: motion artifact in a hyperalgesic child, inadequately premedicated, who bent over due to pain during the acquisition

Pain management is one of the core elements in the optimization of imaging examinations (Fig. 8). Unfortunately, children’s pain is frequently underestimated and inadequately treated. The assessment of pain is based in part on validated scales adapted to the age, such as Numerical Rating Scale (NRS), revised Face Legs Activity Cry and Consolability (r-FLACC) scale, revised Premature Infant Pain Profile (PIPP-R), Faces Pain Scale-Revised (FPS-R), and pain word scale [61].

In children, the fear and the pain due to venipuncture are potential causes of failure in diagnostic procedures. Evidence-based guidelines and reviews have been written to identify strategies to reduce painful sensation or distress [40, 62–64]. Multimodal analgesia approaches should be proposed and premedication anticipated.

Commonly used drugs include non-opioids (acetaminophen and non-steroidal anti-inflammatory drugs, analgesic cream), opioids, anti-spasmodics, muscle relaxants, or neuropathic pain treatments. Nitrous oxide is also an attractive agent for short sedation and analgesia. Non-pharmacological options should also be considered to reduce pain, such as massage, heat compresses, ice packs, and repositioning. When medications are used, it is demonstrated that the combination of two methods, pharmaceuticals (e.g., topical liposomal lidocaine cream) and adjuvants (e.g., instant topical anesthetic skin refrigerant or oral sucrose), is more effective for pain management compared to only one method [40].

Cognitive behavioral strategies are very effective in reducing pain and improving patient compliance. A recent systematic review has described the efficacy of psychological approaches such as distraction, combined cognitive behavioral therapy, and breathing interventions [64]. Parents may function as “coaches” for cognitive behavioral strategies, giving children encouragement for coping mechanisms [25, 65]. However, for this process to be effective, the active participation of parents in the pre-procedure of the NM examination is essential [65].

### Sedation

The decision to sedate a child must be made on an individual basis. Technological developments of recent years have brought about a revolution in NM practices. Increasingly more efficient examinations are available, with less radiation exposure and shorter duration. The use of sedation has become less frequent, and many departments have even abandoned it [4, 8, 66–68]. On the other hand, PET/MR imaging may require sedation, especially in children older than 3–6 months and younger than 6 years, because MR sequences can significantly increase the duration of the examination [69]. When sedation/general anesthesia is required, it will be administered after tracer injection and before image acquisition, especially when performing a brain ^18^F-FDG PET/CT (PET/MRI) due to the effects of anesthesia on regional cerebral glucose metabolism [70].

The depth of sedation is classified as follows: minimal sedation (anxiolysis), moderate sedation (conscious sedation), deep sedation, and general anesthesia [55]. Acute complications are sparse (0.4%), but long-term neurotoxicity and cognitive side effects remain unknown [71]. Some studies reported a higher incidence of adverse events in younger patients with several diseases. Risks of developmental and behavioral disorders and language acquisition issues could be more frequent in children who underwent general anesthesia under 3 years [72].

In the rare cases in which sedation is needed, a multidisciplinary experienced team with an anesthetist is required. To offer sedated examinations, the imaging department must have sufficient human and technical resources (monitoring device, emergency cart, and age-appropriate resuscitation equipment) [8, 55]. Propofol, Dexmedetomidine, Ketamine, Midazolam, and Etomidate are the most used drugs.

Administration of radioactivity in nuclear medicine has medico-legal ramifications. It implies that all efforts should be applied to obtain adequate acquisition of the diagnostic images. The need for sedation and anesthesia should be evaluated in each child prior to the radiopharmaceutical injection. Indeed, the failure to acquire the image within a timeframe consistent with biodistribution, or even inability to acquire it at all, unbalances the risk–benefit assessment that was initially conducted to justify the procedure.

Fasting times for anesthesia should also be considered in the patient preparation. The European Society for Paediatric Anaesthesiology, the American Society of Anaesthesiologists, and the multidisciplinary International Committee for the Advancement of Procedural Sedation propose consensus statements [55, 73]. The American Society of Anaesthesiologists recommend a minimal fasting time of 2 h (h) for clear liquids, 4 h for breast milk, 6 h for infant formula, 6 h for nonhuman milk, 6 h for a light meal, and 8 h or more for fried foods, fatty foods, or meat for gastric emptying.

## Hybrid imaging and CT dose optimization

From the first developments of hybrid imaging, the additional radiation exposure due to CT was a significant concern. Indeed, exposure levels were much higher in the early years. Chawla et al. [74] reported in their study that, from 2002 to 2007, effective doses due to PET/CT ranged from 2.7 to 54.2 milliSieverts (mSv) for the CT scanning and from 0.4 to 7.7 mSv for the PET procedure [75]. Some authors [76–79] reported that a non-optimized CT scan can contribute up to 80% of the whole radiation burden in paediatric NM exams. In this context, the *Image Gently*’s slogan seems more important than ever: “*One size does not fit all*” [80].

In the first instance, the nuclear medicine physician must identify for each patient the body volume to be investigated during the PET/CT or SPECT/CT examination to lower the radiation burden [4]. The physician should also specify whether to include arms in the scanning field of view. The interposition of arms in the field of view often leads to a significant deterioration of image quality and to an increase in the radiation level to compensate for noise [81].

The ALARA principle must always be considered and put into perspective with the benefit-risk ratio of the examination. The optimization of CT in hybrid imaging is mainly based on adjusting acquisition parameters [82–88] as well as reconstruction algorithms [89, 90]. Imaging tests and subsequent standardization of the most common protocols used in the department can help reduce patient exposure [76, 91] and quantify any bias that may be introduced using a very low dose CT for attenuation correction [92, 93].

In SPECT/CT, reducing the CT acquisition volume to the region with the SPECT findings can markedly reduce the radiation dose.

Performing paediatric studies on modern cameras with high detector sensitivity (e.g., cadmium-zinc-telluride (CZT) detectors) and advanced CT scanners can reduce the effective dose by facilitating the lowering of injected activity and using the advanced paediatric CT optimization techniques [94–96]. Similarly, SPECT or PET reconstruction with resolution recovery algorithms can improve image quality and allow reduction in the administered radiopharmaceutical activity [97–100].

Configuration of CT scan parameters for paediatric protocols usually involve optimization of both tube voltage and current, for which weight-based classes for paediatric patients can be defined [82, 89]. Recently, automatic exposure control systems utilizing automatic tube current modulation (ATCM) [83, 87] and automatic tube voltage selection (ATVS) have been developed. ATVS allows an the automatic choice of kV and mAs settings without impairing the contrast-to-noise ratio [101, 102] and negligible variation when used for quantification of PET images [103]. However, exposure control settings will generally still require some weight-based classes to be defined. When the ATVS are not available, tube voltage can be manually adapted for children. Due to the small physical size of children, dose reduction can actually improve image contrast [103], rather than create the severe image noise associated with adult CT.

CT reconstruction algorithms are also crucial for dose reduction [104, 105], and novel reconstruction tools are continuously developed. Several iterative reconstruction methods have been implemented through the years and introduced in NM and PET hybrid systems [106–113]. Furthermore, recent advances in artificial intelligence, using deep learning reconstruction [114, 115], are promising in terms of additional dose reduction and image quality, allowing further adjustment to protocols for specific clinical cases [116].

Therefore, the NM technologist must be aware of the dose reduction options available on their systems and understand how to optimize them properly.

The purpose of the CT will largely determine the CT settings and the radiation exposure to the child. Certain departments prefer the “one-stop-shop” approach, especially with PET/CT utilizing a contrast-enhanced diagnostic CT, which negates the need for additional radiological studies. Nevertheless, since many children undergo diagnostic CT or magnetic resonance imaging (MRI) in addition to the NM procedure, it is common practice to use a low-dose CT settings for anatomical localization and attenuation correction. This is an essential element that must be considered when undertaking a CT optimization study in comparison to that in the radiology field. Examples of the CT optimization in the nuclear medicine field applied to paediatric patients for the purpose of anatomical localization and attenuation correction and the suggested exposure setting used are reported in Table 1.

**Table 1:** The reported nuclear medicine studies had purpose in CT optimization in paediatric patients, aiming at attenuation correction and anatomical localization. *ASiR™*, adaptive statistical iterative reconstruction algorithm; *NI™*, noise index

Authors	Phantom patients	Hybrid system	CT slices	Patient weight (kg^1^)	Tube potential (kVp^2^)	Tube current (mA^3^)	Additional parameters	Tube rotation (s^4^)	Pitch	Slice thickness recon (mm^5^)
Bahn YK et al. [117]	**∙** 10-year-old paediatric equivalent acrylic phantom **∙** NEMA PET phantom™	PET/CT (a)	16	_	80–100	10–40	_	0.5	1.35	3.75
		PET/CT (b)	40	_	80–100	10–40	_	0.19	1.35	5.00
		PET/CT I	16	_	80–100	10–40	_	0.5	1.35	3.75
Brady SL et al. [89]	**∙** Catphan 700 phantom™ **∙** Jaszczak phantom with Esser lid phantom ™ **∙** NEMA IEC Body Phantom™ **∙** 140 patients	PET/CT	64	0–9.4	80	20–130	NI™: 30 ASiR™: 100%	n.a	0.98	3.75
				9.5–18.4	100	20–160	NI™: 40 ASiR™: 100%			
				18.5–31.4	100	10–210				
				31.5–55	100	25–210				
				> 55	120	32–210				
Alkhorayef M [77]	**∙** 27 patients	PET/CT	128	3.1–39	100	20–40	ASiR™: n.a	0.5	1.3	n.a

Legend: ^1^ kg, kilogram; ^2^kVp, kilovoltage peak; ^3^ mA, milliampere; ^4^ s, seconds; ^5^ mm, millimeters

### Effective dose estimation

Workstations [118] provide details of expected and delivered exposure in the form of the volume CT dose index (CTDIvol), representing the dose that would be delivered through a slice of a standard phantom (unit: milliGrays, mGy) and the dose length product (DLP = product of CT dose index and the irradiated scan length, expressed in mGy*centimeter, cm). However, the estimated individual patient risk due to the radiation burden is provided by the effective dose (ED), not by the DLP, and its estimation is a complex process [119, 120] that takes into account the CT parameters, scan range [121, 122], patient size and age, the irradiated organs, body composition, and tissue weighting factors [5].

Nevertheless, in impromptu situations, the DLP can be used indirectly to ascertain the safety of the CT and identify when an exposure may result in higher doses than expected [123].

As a practical example, in PET/CT imaging, the NM technologist can evaluate any unnecessary exposure using the DLP and CTDIvol, displayed before the scan, by comparing these values to reference ranges defined for the weight of the patient.

This check helps increase the awareness of the CT exposure that the NM technologist is using. This strategy does not substitute dosimetry, which should be carried out in an audit setting and is based on precise calculations with dedicated software, usually by the Medical Physics Expert.

### Image review

After image acquisition is complete, a quality assessment of the raw data should be perfomed to confirm the validity of the available images for diagnostic interpretation.

Patient motion artifacts should be assessed, and if present, correction software should be considered (if available). In some instances, image acquisition may need to be repeated [124].

Checking for normal biodistribution of the radiopharmaceutical can help identify any abnormal attenuation artifacts caused by an external object that was inadvertently kept by the patient. In this case, if the attenuation is in the target zone, image acquisition should be repeated.

Patient position artifacts should also be checked, as asymmetrical uptake can be misleading in cases where the patient is not properly positioned. When children are very small, minor rotation may still affect the acquired image and can lead to misinterpretation. Use of post processing and reconstruction methods that allow reorientation might be feasible, else a repeat scan should be considered if a software option is not effective.

It is equally important to check for potential registration artifacts, since both studies — SPECT/PET and CT — should perfectly overlap. Regardless of the need of CT for attenuation correction and/or anatomical reference, an error in the registration can lead to important pitfalls. Again, software and reconstruction solutions should be used when available, and manual reorientation of the scans attempted.

All image repetition should be carefully considered, especially when hybrid imaging is used. Repeating a CT scan will increase the radiation burden of the procedure and should be avoided whenever possible. If only the emission image was affected, repetition is relatively easy to perform, provided the patient is still cooperative. It is useful for a department to audit the frequency that repeat images are required as this may highlight where protocols and practice, particularly related to patient cooperation, can be improved.

### Recommendations on patient discharge

By the end of the procedure, the patient and caregivers should be discharged in friendly manner, to leave a good lasting memory for the child. NM professionals should inform the child pleasantly that the examination is over, orally reinforce the child’s cooperative behaviors, and offer the previously agreed-upon reward. Younger children are often delighted with bubbles, a colored sticker, a fun activity with the caregiver, or the opportunity to put their handprint on the board of the bravest placed in the waiting room. Older children usually feel rewarded with bravery certificates or stickers, given the opportunity to take a photo of the bravest board after having written their name on it. Giving a positive reinforcement to the paediatric patient can be very beneficial to increase his/her well-being and for future examination needs. Parents should also be thanked for cooperating during the procedure and informed about examination results.

Recommendations to stimulate the radiopharmaceutical biological elimination should be given to the patient and/or caregiver. This is obviously dependent on the radiopharmaceutical and its specific excretion. The majority of the radiopharmaceuticals used in NM have renal excretion, so increased hydration and micturition throughout the day are some of the most common final recommendations. Caregivers should be advised to replace diapers more often to reduce radiation exposure.

## Conclusion

Collaboration with the paediatric patient and their caregivers is very challenging and demands specific skills and competencies from the NM professional. Those skills are essential to facilitate a better patient’s experience as well as to avoid complications throughout the procedure. To ensure the highest quality of the procedure and to maintain the comfort of paediatric patient, diagnostic management should be carefully tailored by healthcare providers. “Tailoring” can be understood as careful and thoughtful procedure planning: from appropriate scheduling to discharging the children from the department. Appropriate tailoring reflects the NM professionals’ engagement and helps to ensure and maintain the comfort of children and their caregivers,thereby improving efficacy of the procedure.

### Liability statement

This recommendation summarizes the views of the EANM Technologist, Paediatric and Dosimetry Committees. It reflects recommendations for which the EANM cannot be held responsible. The recommendations should be taken into context of good practice of nuclear medicine and do not substitute for national and international legal or regulatory provisions.
